# Suicidal ideation, attempt and associated factor among secondary school students in Harari regional state, Eastern Ethiopia. A multi-center cross-sectional study

**DOI:** 10.3389/fpsyt.2023.1069910

**Published:** 2023-05-05

**Authors:** Tilahun Bete, Abdi Birhanu, Abraham Negash, Elias Yadeta, Magarsa Lemi, Tegenu Balcha, Addisu Sertsu, Bekelu Birhanu, Shambel Nigussie, Kabtamu Gemechu, Fentahun Meseret, Hanan Mohammed, Addisu Alemu, Deribe Bekele Dechasa, Haftu Asmerom, Mesay Arkew, Abayneh Shewangizaw, Ahmed Mohamed, Fila Ahemed, Dawud Wodaje, Yadeta Dessie, Adera Debella, Tamirat Getachew, Kabtamu Nigussie, Addis Eyeberu

**Affiliations:** ^1^School of Nursing and Midwifery, College of Health and Medical Sciences, Haramaya University, Harar, Ethiopia; ^2^School of Medicine, College of Health and Medical Sciences, Haramaya University, Harar, Ethiopia; ^3^School of Public Health, College of Health and Medical Sciences, Haramaya University, Harar, Ethiopia; ^4^School of Pharmacy, College of Health and Medical Sciences, Haramaya University, Harar, Ethiopia; ^5^School of Medical Laboratory Sciences, College of Health and Medical Sciences, Haramaya University, Harar, Ethiopia; ^6^School of Nursing and Midwifery, College of Health and Medical Sciences, Debre Birhan University, Debre Berhan, Ethiopia

**Keywords:** suicidal behavior, suicidal ideation, suicidal attempt, associated factor, secondary school, adolescents, Harar, Ethiopia

## Abstract

**Background:**

Suicide is a major public health issue across the globe. It is the second leading cause of death in adolescents. Even though the rate of suicide has increased, no study has been conducted to investigate the determinants of suicide in the study area. Therefore, this study aimed to assess the magnitude of suicidal ideation, suicide attempts, and its associated factors among secondary school students in the Harari regional state of Eastern Ethiopia.

**Methods:**

An institutional-based cross-sectional study was conducted among randomly selected 1,666 secondary school students. A structured-self-administered questionnaire was used for data collection. The WHO Composite International Diagnostic Interview (CIDI) was used to assess suicidal ideation and suicide attempts. The Depression Anxiety and Stress Scale (DASS) was also used to assess depression, anxiety, and stress. Data were entered into EpiData version 3.1 and exported to Stata version 14.0 for the analysis. A logistic regression analysis was performed to determine the association between the outcome and independent variables and the statistical significance was declared at a *p*-value of < 0.05.

**Result:**

The overall magnitude of suicidal ideation and attempts was 13.82% at 95% confidence interval (CI): 12.16–15.66 and 7.61% at 95% CI: 6.37–9.07, respectively. Suicidal ideations and suicide attempts were significantly associated with undergoing depressive symptoms (adjusted odds ratio [AOR]: 1.54; 95% CI: 1.08–2.19 and AOR: 2.37; 95% CI: 1.46–3.86, respectively), experiencing anxiety symptoms (AOR: 1.80; 95% CI: 1.25–2.59 and AOR: 1.89; 95% CI: 2.14–10.65, respectively), being exposed to sexual violence (AOR: 3.36; 95% CI: 1.65–6.84), and having a family history of suicidal attempts (AOR: 2.12; 95% CI: 1.21–3.69 and AOR: 4.74; 95% CI: 2.14–10.65, respectively), whereas living in a rural residence (AOR: 1.65 95%, CI: 1.08–2.55) was significantly associated only with suicide attempts.

**Conclusion and recommendations:**

Nearly one in six secondary school students had both suicidal ideation and attempted to take their own life. Suicide is one of the psychiatric emergencies that need immediate action. Therefore, the concerned body from either a governmental or a non-governmental organization should work in setting strategies to minimize sexual violence as well as depressive and anxiety symptoms.

## Introduction

Suicide is the deliberate act of one's death (from the Latin suicaedere, “to kill oneself” or “self-murder”). When there is proof (either explicit or implicit) that the injury was self-inflicted as well as the decedent meant to take him or her own life, it is considered a self-inflicted injury, poisoning, or suffocation death. A suicide attempt is a non-habitual act with a non-fatal outcome that is intentionally started and carried out by the person involved, causes self-harm, or will be carried out without assistance from others, or involves ingesting a substance higher than the generally accepted therapeutic dosage (1–4). Suicidal ideation is any self-reported passive desire to die or an active desire to take their own life without accompanying preparatory behavior (2, 4).

Suicide is the second leading cause of death in youths worldwide, accounting for notably 1.4% of the global disease burden. Suicide claims more lives annually than all homicides and all wars combined. Although suicide rate increases with age, suicidal behavior is common and on the rise among young individuals between the ages 15 and 25 years. In this age bracket, each year, more than 800,000 teenagers die by suicide (5–7).

There is a growing body of literature that examines this health hazard in low- and middle-income countries, even though the majority of research on suicidal ideation and attempts among adolescents has been done in high-income countries. Evidence showed that suicidal ideation and attempts are very common across different settings (8, 9). The lifetime suicidal ideation rates ranged from 6% in India (10) to 25.28% in Palestine (11). The prevalence of suicide attempts varied among countries, ranging from 0.39% in India (10) to 2.7% in China (12) and to 3.8% in Vietnam (13).

In sub-Saharan Africa, death from suicide is estimated to be 34,000 per year (14). It has been observed in various studies that high rates of secondary school students considered suicide in 2022. Estimates of the prevalence of suicidal ideation in Africa ranged between 6.25% and 31.3% (15–21). The magnitude of suicide attempts ranges between 2.8% and 17.3% (21–23).

Different biological, psychological, and social factors, such as, being a female, loneliness, depression, anxiety, and psychoactive substance use, alcohol consumption, the feeling of hopelessness, school absenteeism, poor social support, and physical hurt, are related to suicidal ideation and suicide attempts (15, 16, 18–20, 23–25). In the continent of sub-Saharan Africa, attempts at take their own life among young people aged 15 to 24 years have been reported to be very common, with suicidal rates ranging in the study population from 12.3% in Southwest Nigeria to 28.3% in Benin in 2022 (19, 24, 26). Information on the causes of teen suicide attempts in low-income countries is, surprisingly, scarce. According to a study conducted in China (12), parental reprimand, punishment, and family gambling were all linked to suicide attempts. In Tanzania, suicide attempts were correlated with loneliness, depression, cigarette usage, and a lack of friends (15).

Suicide is a serious, curable public health issue that affects friends, family, and coworkers negatively on social, emotional, and financial levels (1, 5). Despite the difficulty involved in its prevention and control, suicide demands our attention and action (27, 28). Despite this sporadic research study, the full scope of the issue in Ethiopia is poorly understood because there have been very few data and statistics available of the country on suicide and are not well-understood (23, 29). This study makes a start toward filling this gap by estimating the prevalence and contributing factors of suicidal ideation and suicide attempts among secondary school students. Furthermore, this study identifies the role of having a history of sexual abuse on the suicidal impulses. This study will contribute valuable data for decision-making authorities and policymakers, health professionals, and other concerned stakeholders who would like to apply some intervention mechanisms regarding the issue. Moreover, the study will help the participants or the students to gain attention and knowledge about suicide. Furthermore, this study can be used as a baseline for future researchers who would like to undertake further investigation and systematic review on the subject. Therefore, this study aimed to assess the magnitude of suicidal ideation, suicide attempts, and their associated factors among secondary schools in the Eastern Ethiopia.

## Methods and materials

### Study setting, design, period, population, and eligibility

A school-based cross-sectional study was carried out in the Harari regional state of Eastern Ethiopia from 10 April 2022 to 10 May 2022. Harar is the capital city of the Harari regional state of Eastern Ethiopia. The city also serves as the East Hararge Zone's administrative center in the Oromia region and is located at an altitude of 1,885 m (6,184 feet)—around 526 km from Ethiopia's capital city Addis Ababa (30). The population (2021 projection based on the 2007 Census, Central Statistical Agency [CSA]) of the region was estimated to be 270,000, from which 136,000 were boys/men and 134,000 were girls/women. There are 10 public secondary schools in the region. More than 9,589 students were enrolled in those government schools in 2022. Of those students, 43.3% were girls. All secondary school students of the Harari regional state were the source population, and all randomly selected students among the source population were the study population. Students who were available during data collection from randomly selected schools were included in the study. Students who were seriously ill during the data collection period were excluded.

### Sample size determination and sampling procedure

The sample size was calculated by using a single population proportion formula with the following statistical assumptions: *n* = the minimum sample size required, *p* = the estimated proportion of common mental disorders, *Z* = the standard value of confidence level of α = 95%, and *d* = the margin of error between the sample and the population (0.01). For this study, *p* = 12.5% (the prevalence of suicide attempts from Fitche town in Ethiopia) was used as reference (25).


$$\begin{array}{lll} n & = & \frac{{\left(\;Z_{\frac{\alpha }{2}}\right)}^{2}\;p\;(1-p)}{d^{2}}\\ n & = & \frac{{\left(\;1.96\right)}^{2}\;0.125\;(1-0.125)}{{\left(0.02\right)}^{2}}=\;1,050 \end{array}$$

**Figure 1:**
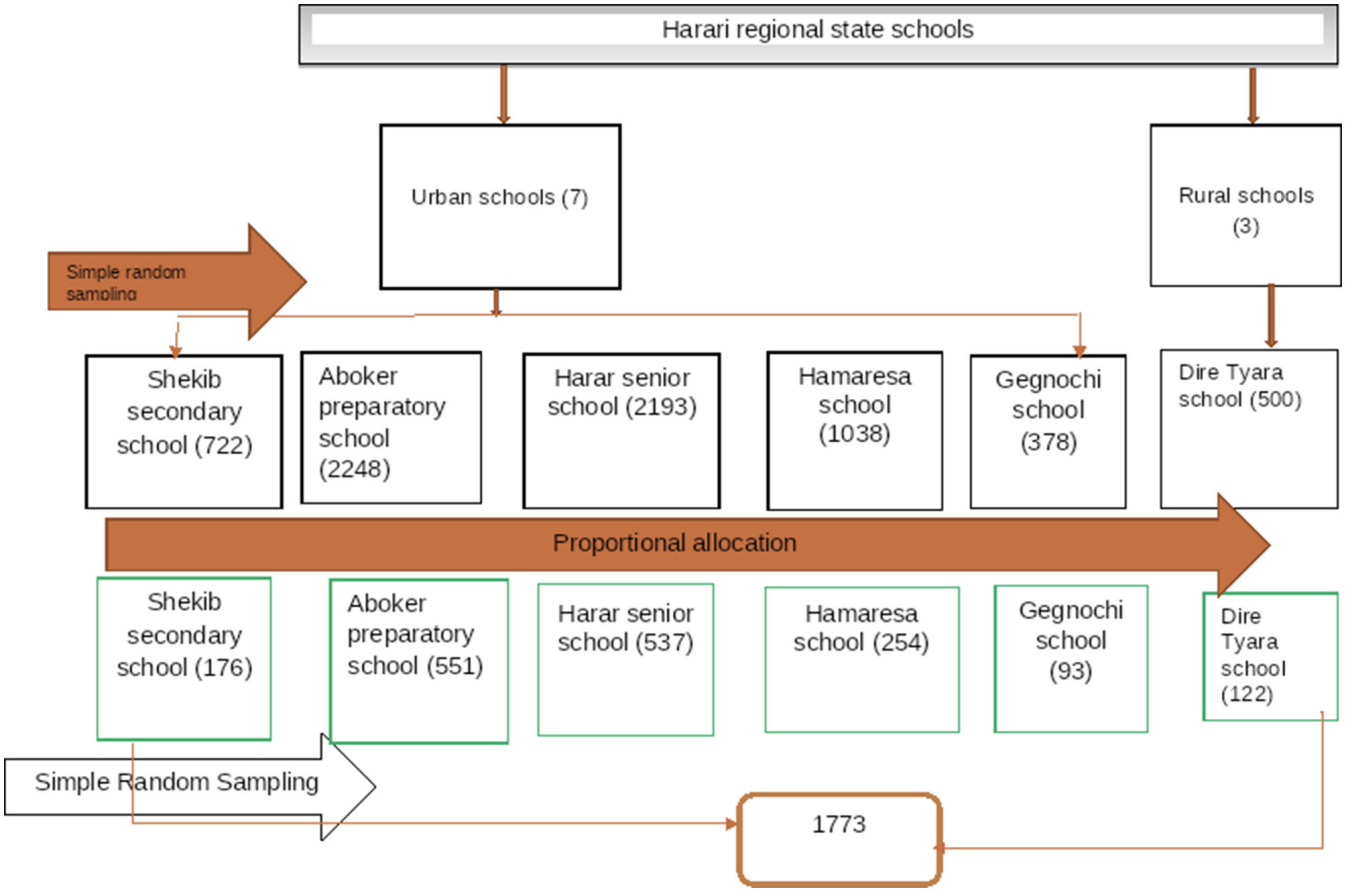
The schematic diagram for the sampling procedure of the assessment of suicidal ideation, attempt, and associated factors among secondary school students in the Harari Region in Eastern Ethiopia.

Accordingly, with a design effect of 1.5 and adding a 10% non-response rate, the final sample size was measured as 1,733. There are 10 government secondary schools in the region. A total of 6 secondary schools were selected randomly from those 10 schools. Then, the number of students in each selected school was included, and proportional allocation was done for each selected secondary school. The total number of students in those randomly selected schools was 7,079. Finally, simple random sampling was used to select the study participants by using registration as a sampling frame (Figure 1).

### Data collection tools

The data were collected through a face-to-face interview with a pretested, structured, and standard questionnaire that was adopted by reviewing recent literature (15, 18, 20). The questionnaires inquired about participants' socioeconomic and demographic information, depression status, anxiety status, stress status, substance use status, social support status, history of sexual violence, suicidal ideation, and suicide attempts. Depression, anxiety, and stress were assessed by adopting the Depression Anxiety Stress Scale 21 (DASS-21) questionnaire. DASS-21 is a validated and reliable instrument with 21 items in three domains. Each domain comprises seven items. Scores from each dimension were added and multiplied by two. Those who scored above 0 for depression were considered normal, and those who scored above 9 were considered a case of depression. Those who scored above 8 and those who scored above 15 were considered to having anxiety and stress, respectively (28–30). DASS-21 has excellent Cronbach's α values of 0.81, 0.89, and 0.78 for the subscales of depression, anxiety, and stress, respectively (30). The suicide module of the World Mental Health (WMH) survey, Composite International Diagnostic Interview (CIDI), an initiative of the World Health Organization (WHO), was used to assess suicidal ideation and suicide attempts. The tool was validated in Ethiopia in both clinical and community settings (31). The Oslo 3-item Social Support Scale was used to assess social support. A sum index was made by summarizing the raw scores, where the sum ranged from 3 to 14, which was reliable in the study (Cronbach's α = 0.91) performed in Wolayta University (32, 33). A total of 20 nurses with a Bachelor of Science degree collected the data after a 5-day training session on the tools and survey methods. The Pittsburgh Sleep Quality Index (PSQI) was used to assess sleep quality among students that contain 19 items classified into 7 components: each component scores ranged from 0 to 3 and then a global score with an interval from 0 to 21 was obtained. A global score >5 was considered poor sleep quality, and scores ≤5 were considered good quality sleep (34).

### Operational definitions

#### Suicidal ideation

If the respondent answers the question “Have you seriously thought about taking your own life in the last 12 months?”, with the answer “yes,” then it is considered that the respondent had suicidal ideation.

#### Suicide attempt

If the respondent answers the question “Have you attempted to take your own life in the last 12 months?” with the answer “yes,” then it is considered that the respondent had attempted to take her/his own life.

#### Current substance use

Students who are currently using or who used at least one of the substances (alcohol, khat, cigarette, etc.) in the past 3 months were considered as current substance users.

#### Sexual abuse

Those individuals who encountered forceful sexual and physical abuse were considered as having sexual abuse.

#### Social support

Using the Oslo Social Support Scale, those students who scored < 3–8 on the scale were considered as having low social support, 9–11 were considered as having medium social support, and 12–14 were considered as having strong social support.

#### Anxiety symptoms

Students who scored ≥8 in the DASS-21 score were considered as having anxiety symptoms (32).

#### Depressive symptoms

Students who scored ≥10 in the DASS-21 score were considered as having depressive symptoms (32).

#### Sleep quality

Students who scored PSQI ≤ 5 were considered as having good sleep quality (34).

#### Stress

Students who scored on DASS-21 “≥15” were declared to having stress (32).

#### Sexual abuse

Sexual abuse was assessed based on “Yes” or “No” questions, such as “Have you ever been sexually abused?”, if they said “Yes,” they were considered to having experienced sexual abuse.

### Data quality control

The questionnaire was initially prepared in the English language and then translated into the local languages by a bilingual expert (Afaan Oromoo and Amharic language). Then, the questionnaire was translated back into the English version to ensure its consistency. The data collectors and field supervisors received training on the data collection tool and procedures. Before the data collection of the study, the pretest was conducted among 10% of secondary school students in the secondary schools of the region out of the study area, namely, in the districts of Haramaya, Harar, Ethiopia. The investigators and experienced field research supervisors provided regular supervision.

### Data processing and analysis

First, the collected data were checked for completeness and consistency. The data were then cleaned, coded, and entered into EpiData version 3.1 for further analysis. The entered data were exported to Stata version 14.1 for analysis. The outcome variable was recoded into binary outcomes as “yes = 1” and “No = 0”; variables with a *p*-value of < 0.2 in the bivariable analysis were entered into multivariable logistic regression analysis (33). A multivariable logistic regression analysis was performed to identify the factors associated with the outcome variable. The goodness-of-fit model was checked by using the Hosmer-Lemeshow test. The direction and strength of statistical association were measured by odds ratio (OR) along with the 95% confidence interval (CI); a *p*-value of < 0.05 was considered statistically significant both in bivariable and in multivariable analyses.

## Results

### Sociodemographic characteristics of respondents

Among 1,733 eligible participants, 1,615 have participated in the study, which gives a response rate of 93.19%. The mean age of the respondents was 17.31 years with a standard deviation (SD) of ±2.08. More than half of the participants were girls (880 [54.49%]), and most of them followed the Islamic religion (1,112 [68.85%]). Regarding their educational status, nearly half of the students were in grade 9 (Table 1).

**Table 1:** Sociodemographic characteristics among secondary school students at Harari region state public schools of Eastern Ethiopia (*n* = 1,615).

**Variables**	**Categories**	**Frequency (*n* = 1,615)**	**Percentage (%)**
Sex	Boys	735	45.51
	Girls	880	54.49
Age	< 14	15	0.93
	14–19	11,399	86.62
	>19	201	12.45
Marital status	Single	1,231	76.22
	In-relationship	320	19.81
	Married	64	3.96
Religion	Muslim	1,112	68.85
	Orthodox	396	24.52
	Protestant	89	5.51
	Catholic	11	0.68
	Other	7	0.44
Education	Grade 9	767	47.49
	Grade 10	579	35.85
	Grade 11	243	15.05
	Grade 12	26	1.61
Place of residence	Urban	1,091	67.55
	Rural	524	32.45

Other in religion includes Adventist and waqqefeta.

### Substance use, mental illness, and psychosocial characteristics of the respondents

Among the total respondents, 102 (6.81%) had a family history of mental illness, and 83 (5.54%) of them had a family history of suicide attempts. Nearly one-quarter of the participants were current khat users. Nearly half of the participants had poor social support 716 (47.80%). More than one-third of the participants (677 [41.92%]) have depressive symptoms and 655 (40.56%) of them have anxiety symptoms. Nearly 41 (2.54%) study participants had a history of sexual abuse (Table 2).

**Table 2:** Clinical, chat use, psychosocial characteristics among secondary school students at Harari region state public schools of Eastern Ethiopia (*n* = 1615).

**Variables**	**Categories**	**Frequency (*n* = 1,615)**	**Percentage**
Family history of mental illness	Yes	110	6.81
	No	1,505	93.19
Family history of attempts to take their own life	No	1,531	94.80
	Yes	84	5.20
Sleep quality	Good sleep quality	1,138	70.46
	Poor sleep quality	477	29.54
Current chat user	No	1,261	78.08
	Yes	354	21.92
Ever sexually abused	No	1,574	97.46
	Yes	41	2.54
Social support	Poor social support	772	47.8038.27
	Medium social support	618	13.93
	Strong social support	225	
Depression	No	938	58.08
	Yes	677	41.92
Anxiety	Yes	960	59.44
	No	655	40.56
Stress	No	1,265	78.33
	Yes	350	21.67

### The magnitude of suicidal ideation and suicide attempts among respondents

The overall magnitude of suicidal ideation and suicide attempt among the secondary school students for the last 12 months was 218 (13.46%) at 95% CI (11.92–15.25) and 125 (7.74%) at 95% CI (6.53–9.15), respectively. The lifetime suicidal ideation and suicide attempt were 19.5% and 9.89%, respectively. The most common methods of suicide attempt were using poison (65% of the students) and hanging (25% of the students).

### Factors associated with suicidal ideation among secondary school students

Variables selected for multivariable logistic regression analysis were based on their *p*-value less than 0.20. In a multivariable logistic regression analysis for depressive symptoms, anxiety symptoms, a family history of suicide attempts, and ever being sexually abused were significantly associated with suicidal ideation at a *p*-value of < 0.05 at 95% CIs.

Individuals who have encountered sexual violence were (AOR = 3.49; 95% CI: 1.75–7.02) more likely to have suicidal ideation more than three times than those who have not been sexually encountered. Individuals who have a family history of suicide attempts were (AOR =2.02; 95% CI: 1.16–3.53) more likely to have suicidal ideation by more than two times than those who have no family history of suicide attempts.

The odds ratios of having suicidal ideation among participants who have depressive and anxiety symptoms were 1.44 and 1.88 times higher as compared to those who have no depressive and anxiety symptoms ([AOR =1.44; 95% CI: 1.02–2.03] and [AOR = 1.88; 95% CI: 1.32–2.03], respectively) as shown in Table 3.

**Table 3:** Factors associated with suicidal ideation among secondary school students attending Harari regional state schools of Eastern Ethiopia (*n* = 1,615).

**Variable**	**Variable classification**	**Suicidal Ideation**		**COR (95%CI)**	**AOR (95%CI)**	***P*-value**
Gender	Boys	631	104	1	1	1
	Girls	766	114	0.90(0.68, 1.20)	1.11(0.80–1.52)	0.536
Marital status	Single	1,079	152	0.51(0.27, 0.93)	0.54(0.28–1.02)	0.057
	In-relationship	268	52	0.69(0.36, 1.34)	0.75(0.37–1.48)	0.403
	Married	50	14	1	1	1
Social support	Poor	667	105	0.82(0.55, 1.25)	0.76(0.49–1.17)	0.213
	Medium	541	77	0.75(0.49, 1.15)	0.72(0.46–1.13)	0.149
	Strong	189	36	1	1	1
Depression symptoms	No	845	93	1	1	1
	Yes	552	125	2.06(1.54, 2.75)	1.44(1.02–2.03)	**0.037** ^*^
Anxiety symptoms	No	872	88	1	1	1
	Yes	525	130	2.45(1.83, 3.28)	1.88(1.32–2.67)	**0.001** ^*^
Stress	No	1,117	148	1	1	1
	Yes	280	70	1.89(1.38, 2.58)	1.11(0.77–1.59)	0.582
Family history of mental illness	No	1,311	194	1	1	1
	Yes	86	24	1.89 (1.17, 3.04)	1.26(.74–2.17)	0.397
Family history of attempt to take their own life	No	1,337	194	1	1	1
	Yes	60	24	2.76(1.67, 4.53)	2.02(1.16–3.53)	**0.013** ^*^
Sleep Quality	Good	998	140	1	1	1
	poor	399	78	1.34(1.03, 1.88)	1.05(0.75–1.46)	0.782
current chat user	No	1,103	158	1		
	Yes	294	60	1.42(1.03, 1.97)	1.39(0.97–1.99)	0.075
Ever sexually abused	No	1,372	202	1	1	1
	Yes	25	16	4.35(2.28, 8.28)	3.49(1.75–7.02)	**< 0.001** ^*^

COR, Crude odds ratio; AOR, Adjusted Odds ratio; CI, Confidence interval; 1, reference Hosmer-Lemeshow test 0.713. ^*^Statistically significant variable at *p*-value of less than 0.05.

### Factors associated with suicide attempts among secondary school students

Variables selected for a multivariable logistic regression analysis were based on their clinical examination outcomes and a *p*-value of < 0.20. In a multivariable logistic regression analysis, having depressive symptoms, anxiety symptoms, having a family history of suicide attempts, having encountered sexual violence, living in urban areas, and being a female were significantly associated with suicidal ideation at a *p*-value of < 0.05.

Females were 1.59 times (AOR = 1.59; 95% CI: 1.05–2.42) more likely to have suicidal attempts than males. The odds ratio of suicide attempts were 1.65 times (AOR = 1.62; 95% CI: 1.08–2.43) more likely to occur among rural residents than among urban residents.

Individuals who encountered sexual violence were more than four times (AOR = 4.77; 95% CI: 2.19–10.38) likely to attempt to take their own life than those who had never encountered sexual violence. Individuals who have a family history of suicide attempts were more than 2 times (AOR = 2.28; 95% CI: 1.18–4.39) likely to attempt to take their own life than those who have no family history of attempts to take their own life.

The odds ratios of suicide attempts by participants who have depressive and anxiety symptoms were 1.96 and 2.09 times higher as compared to those who have no depressive and anxiety symptoms (AOR = 1.96; 95% CI: 1.25–3.09) and (AOR = 2.09; 95% CI: 1.31–3.34), respectively, as shown in Table 4.

**Table 4:** Factors associated with suicidal attempt among secondary school students attending Harari regional state schools, Eastern Ethiopia (*n* = 1,615).

**Variable**	**Variable classification**	**Attempt to take their own life**		**COR (95% CI)**	**AOR (95% CI)**	***P*-value**
		**No**	**Yes**			
Gender	Boys	682	53	1	1	1
	Girls	808	72	1.15(0.79–1.66)	1.59(1.05–2.42)	**0.030** ^*^
Marital status	Single	1,148	83	0.44(0.21–0.93)	0.53(0.24–1.16)	0.114
	In-relationship	287	33	0.70(0.32–1.55)	0.81(0.35–1.87)	0.618
	Married	55	9	1	1	1
Residence	Rural	472	52	1.54(1.06–2.23)	1.62(1.08–2.43)	**0.019** ^*^
	Urban	1,018	73	1	1	1
poor social support	Poor	715	57	0.77(0.46–1.31)	0.70(0.40–1.24)	0.221
	Medium	571	47	0.79(0.47–1.37)	0.74(0.42–1.32)	0.309
	Strong	204	21	1	1	1
Depression symptoms	No	896	42	1	1	1
	Yes	594	83	2.98(2.03–4.38)	1.96(1.25–3.09)	**0.004** ^*^
Anxiety symptoms	No	919	41	1	1	1
	Yes	571	84	3.29(2.24–4.86)	2.09(1.31–3.34)	**0.002** ^*^
Stress	No	1,188	77	1	1	1
	Yes	302	48	2.45(1.67–3.59)	1.30(0.84–2.03)	0.242
Family history of mental illness	No	1,397	108	1	1	1
	Yes	93	17	2.36(1.36–4.11)	1.42(0.73–2.74)	0.298
Family history of attempt to take their own life	No	1,424	107	1	1	1
	Yes	66	18	3.63(2.08–6.33)	2.28(1.18–4.39)	**0.014** ^*^
Sleep Quality	Good	1,063	75	1	1	1
	Poor	427	50	1.66(1.14–2.41)	1.16(0.76–1.77)	0.479
current chat user	No	1,170	91	1	1	1
	Yes	320	34	1.37(0.90–2.06)	1.36(0.85–2.19)	0.198
Ever sexually abused	No	1,462	112	1	1	1
	Yes	28	13	6.06(3.05–12.02)	4.77(2.19–10.38)	**< 0.001** ^*^

COR, crude odds ratio; AOR, adjusted odds ratio; CI, confidence interval; 1: reference; Hosmer–Lemeshow test: 0.5750. ^*^Statistically significant variable at *p*-value of less than 0.05.

## Discussion

The result of this study revealed that the magnitude of suicidal ideation and suicide attempt was 13.46% (95% CI: 11.92–15.25) and 7.74% (95% CI: 6.53–9.15), respectively. Those who have depressive and anxiety symptoms, who have anxiety symptoms, and who experienced sexual abuse were significantly associated with suicidal ideations whereas those who were rural residents, who have depressive and anxiety symptoms, who had a family history of suicide attempts, and who experienced sexual abuse were significantly associated with suicide attempts.

According to the findings of this study, the prevalence of suicidal ideation was 13.46%. A similar finding was reported in a study conducted in Nepal, which reported the prevalence of suicidal ideation as 13.9% (35). This could be because they use a similar strategy for sampling and have a similar social structure. This result was higher than that of a study conducted in Tunisia with 9.6% (36), Brazil with 7.9% (37), Tanzania with 7% (38), and Nigeria with 6.1% (22). This inequality could be attributed to differences in tools used in assessing suicidal ideation and suicide attempts. For example, the Tunisian study used the Suicide Behavior Questionnaire—Revised and the Brazil, Tanzania, and Nigeria studies used “During the past 12 months, did you ever seriously consider suicidal ideation?”, whereas this study used World Health Organization (WHO) Composite International Diagnostic Interview (CIDI). Another possible explanation is the assessment of the difference in the duration of the suicidal thought; for example, the Nigerian study only assessed suicidal ideation in the past 1 month whereas this study assessed suicidal ideation in the past 12 months. One more reason might be the economic, social, and cultural differences among the population. On the contrary, finding indicate the result of current study was relatively lower than the finding from the studies conducted in Ethiopia Fiche town, 20.5% (39); Northwest Dangila Town, 22.3% (23); Rural Uganda, 21.6 % (20); Fitche town, 20.5%; Swaziland, 18.3%(40); Ghana, 25.1% (21); Benin, 23.2% (19); Peru, 26.3% (41); and Colombia, 33.6% (42). This inequality could be attributed to differences in tools used; for example, the Ghana study used the Suicide Behavior Questionnaire—Revised, whereas this study used CIDI in assessing suicidal ideation and suicide attempts. Further reason might be that the Ghana and the Uganda studies were conducted only in rural area whereas this study included both rural and urban areas. Another possible explanation is the economic, social, and cultural differences among the population.

This study highlighted that the prevalence of suicidal attempts was 7.74%. In line with the study conducted in Tunisia, this finding from this study concerning the prevalence of suicide attempts was 7.3% (36). This could be because they use a similar strategy for sampling and have a similar social structure. The finding from this study was higher than that of the study conducted in Nigeria 2.8% (22). This inequality could be attributed to differences in the duration of the study covered. The Nigeria study only assessed the past 1 month, while this study assessed the past 1 year. In contrast, the finding from this study was relatively lower than that from the studies conducted in Dangila, Ethiopia, 16.2% (23); Fitche, 12.5% (25); Nepal, 10.33% (35); and Peru 17.3% (41). This inequality could be attributed to the differences in the prevalence rates of suicidal behavior found in this study, and those found in other studies of African countries may be due to differences in the meaning of suicidal thoughts and normative attitudes toward suicide across diverse cultural, religious, and economic settings. To put the entire scenario in a nutshell, suicidal ideation and suicide attempt rates appear to vary by country. This is most likely due to the complex and interactive nature of suicidal behavior's underlying factors at the individual, community, and societal levels.

The odds ratios of having suicidal ideation and suicide attempts are more than 1.44 times and nearly 2 times more likely to occur among individuals with depressive symptoms than those without depressive symptoms, respectively. This finding is similar to that of other studies in Vietnamese and English (43–48); the possible reason might be students who have high depressive symptoms may have suicidal ideation. Adolescents with depressive symptoms may have difficulty controlling the situation and may have negative feelings about themselves and the world. They also feel persistently defeated; these feelings may lead them to suicide. Further reason might be a loss of interest by adolescents with depressive symptoms in everything that is the key, which leads the individual to suicide. Depression also causes distress and affects the decision-making aspect of the individual due to those individuals with depressive symptoms may think and attempt to end their life as a means of escape from their feelings (47, 49, 50).

The results of this study showed that anxiety was significantly associated with suicidal ideation and suicide attempts. This finding is similar to those of the Vietnamese and Taiwanese (46, 51). A possible justification might be that students with anxiety symptoms have an excessive feeling of worry. Worrinness is the main and key symptom of anxiety. It refers to the overwhelming or distressing and excessive thoughts about the past, current, and future. Individuals with worry symptoms may worry about themselves, their friends, and their family without having sufficient reason or beyond the reason that possesses students to suicidal ideation and suicide attempt (44). Further reason might be anxiety symptoms that may become overwhelming or distressing to the adolescent, directly inciting thoughts of suicide as a means of escape.

According to this study, those with a family history of suicide attempts were 2.02 and 2.28 times more likely to experience suicidal ideation and suicide attempts, respectively, than their counterparts. This finding is consistent with studies conducted in Brazil (37) and Tanzania (38). This is because evidence suggests that suicide can run in families. Furthermore, numerous studies have consistently shown that family members of victims and those who attempted to take their own life are at a significantly higher risk of suicidal behavior (52–54), which could be due to attempting anniversary suicide.

Furthermore, in this study, sexual abuse was found to be an independent predictor of both suicidal ideation and attempts to take their own life. As a result, students who had encountered sexual violence were 3.49 times and 4.77 times more likely to experience sexual ideation and suicide attempts, respectively. These findings are also supported by studies conducted in Cambodia (42), Peru (41), Ethiopia (23), Ghana (55), Swaziland (40), and Brazil (37). This could be due to the exposure and participation in these harmful behaviors, which may contribute to suicidal behavior through accumulative internalized behaviors such as social isolation, shame, and depression-like feelings, which eventually affect their ability to deal with such stressors associated with abuse (19). These findings highlight the importance of developing an intervention policy aimed at reducing violence-related behaviors among school-aged adolescents (56).

In the final model of multivariable analysis, girls were 1.59 times more likely to attempt to take their own life compared to boys. This finding is in line with previous studies (57–59). The possible justification that girls may attempt to take their own life more than boys is due to having different psychosocial stressors and coping mechanisms for the events. Being encountered with sexual violence and physical violence disturbs emotional stability, which may lead to more girls/women attempting to take their own life (60). Further reason might be the higher number of participations by female participants and sexual victims in our study and thus the increase in suicide among females. Another possible justification might be that boys/men take their own life rather than attempt because of the more fatal method used, whereas girls/women use less fatal methods which thus leads to an increase in attempts at taking their own life among girls/women than boys/male. Further reason with regard to socialization theory on gender differences in suicide is that girls/women are indecisive, dependent, and express their worries or stress through rumination, which leads more encounters of attempts to take their own life by girls/women (61).

Finally, the study highlighted that the participants who were rural residents were l.62 times more likely to suicidal attempt to take their own life than their counterparts. This is consistent with the finding from a previous study (62). The possible explanation is that those who came from rural areas struggle to live in the city because they are exposed to a lot of trouble.

### Strengths and limitations of the study

While the use of standard and validated tools to assess both dependent and independent variables and taking a sufficient sample to represent the target population were considered the strength of the study, despite this study having a cross-sectional study design, the study design does not allow the establishing of a temporal relationship between outcomes and independent variables.

## Conclusion and recommendations

The overall magnitude of both suicidal ideation and suicide attempts was a common problem among secondary school students of the Harari regional state of Eastern Ethiopia compared to other study findings. Regarding the associated factors having depressive symptoms, anxiety symptoms, having a family history of suicide attempts, and being encountered with sexual abuse were significantly associated with suicidal ideation. Being a female, living in a rural residence, having depressive symptoms, anxiety symptoms, having a family history of suicide attempts, and being encountered with sexual violence were significantly associated with suicide attempts. Suicide is one of the psychiatric emergencies that need immediate action. Therefore, the concerned governmental or a non-governmental body should provide better intervention. We would recommend that Harari Regional Health Bureau provide school-based education regarding suicidal behavior with the help of a mental health professional.

## Data availability statement

The original contributions presented in the study are included in the article/supplementary material, further inquiries can be directed to the corresponding author.

## Ethics statement

The studies involving human participants were reviewed and approved by Institutional Health Research Ethics Review Committee (IHRERC) of the College of Health and Medical Sciences of Haramaya University. Written informed consent to participate in this study was provided by the participants' legal guardian/next of kin.

## Author contributions

All authors made a significant contribution to the work reported, which included the conception, study design, execution, acquisition of data, analysis, and interpretation or all these areas, took part in drafting, revising, or critically reviewing the article, gave final approval of the version to be published, have agreed on the journal to which the article has been submitted, agreed to be accountable for all aspects of the work, read the final version of the manuscript, and approved this version of the manuscript to be considered for publication.
